# Dieting practices, weight perceptions, and body composition: A comparison of normal weight, overweight, and obese college females

**DOI:** 10.1186/1475-2891-5-11

**Published:** 2006-03-31

**Authors:** Brenda M Malinauskas, Thomas D Raedeke, Victor G Aeby, Jean L Smith, Matthew B Dallas

**Affiliations:** 1 Department of Nutrition and Hospitality Management, East Carolina University, Greenville, North Carolina, USA; 2 Department of Exercise and Sport Science, East Carolina University, Greenville, North Carolina, USA; 3 Department of Health Education, East Carolina University, Greenville, North Carolina, USA

## Abstract

**Background:**

Of concern to health educators is the suggestion that college females practice diet and health behaviors that contradict the 2005 dietary guidelines for Americans. In this regard, there remain gaps in the research related to dieting among college females. Namely, do normal weight individuals diet differently from those who are overweight or obese, and are there dieting practices used by females that can be adapted to promote a healthy body weight? Since it is well recognized that females diet, this study seeks to determine the dieting practices used among normal, overweight, and obese college females (do they diet differently) and identify dieting practices that could be pursued to help these females more appropriately achieve and maintain a healthy body weight.

**Methods:**

A total of 185 female college students aged 18 to 24 years participated in this study. Height, weight, waist and hip circumferences, and skinfold thickness were measured to assess body composition. Surveys included a dieting practices questionnaire and a 30-day physical activity recall. Participants were classified according to body mass index (BMI) as normal weight (n = 113), overweight (n = 35), or obese (n = 21). Data were analyzed using JMP IN® software. Descriptive statistics included means, standard deviations, and frequency. Subsequent data analysis involved Pearson *X*^2 ^and one-way analysis of variance with comparison for all pairs that were significantly different using Tukey-Kramer honestly significant difference test.

**Results:**

Outcomes of this study indicate the majority of participants (83%) used dieting for weight loss and believed they would be 2% to 6% greater than current weight if they did not diet; normal weight, overweight, and obese groups perceived attractive weight to be 94%, 85%, and 74%, respectively, of current weight; 80% of participants reported using physical activity to control weight, although only 19% exercised at a level that would promote weight loss; only two of 15 dieting behaviors assessed differed in terms of prevalence of use among groups, which were consciously eating less than you want (44% normal weight, 57% overweight, 81% obese) and using artificial sweeteners (31% normal weight and overweight, 5% obese); and the most prevalent explicit maladaptive weight loss behavior was smoking cigarettes (used by 9% of participants) and most unhealthy was skipping breakfast (32%).

**Conclusion:**

Collectively, results indicate female college students, regardless of weight status, would benefit from open discussions with health educators regarding healthy and effective dieting practices to achieve/maintain a healthy body weight. The results are subject to replication among high school, middle-aged, and older females.

## Background

Tens of thousands of high school graduates will be heading out on their own this fall. Many will be confronting the rigors of higher education while adjusting to a new environment and new pressures. With all the changes, gaining weight is common. When you consider that more than 1.5 million students are entering U.S. colleges and universities each fall, by the following spring the weight gain entering collegians encounter is epidemic. Gaining weight during freshman year is so common that the phenomenon has its own nickname the "Freshman 15" and has been associated with the freshman experience for many years. Although unwanted weight gain can occur at any age, the transition from high school to college is a time of increased risk of weight gain among females.

Females, at a very young age, are concerned about body weight and place high importance on appearance, which is dramatically influenced by the media. A major messaging theme of teen magazine websites is the necessity to be beautiful [1]. Mooney et al [2] conducted interviews with home economic teachers and teen focus groups in Ireland and found that adolescent females are very conscious of their body image, are strongly influenced by high profile celebrities, and the primary motivations for wanting to be thin are to gain attention from males, approval from friends, and self confidence. Unfortunately, the desire to be beautiful may result in unwanted outcomes. For example, in a study by Monro et al [3] the authors reported an increase in appearance anxiety among female college students that resulted from viewing advertisements of idealized images. Wilson et al [4] reported that non-athlete college students reported more stress than athlete counterparts in areas of satisfaction with physical appearance, decisions about education, social conflicts regarding smoking, and financial burdens. Lastly, there are unique experiences of college females that may promote dieting, including fear of gaining weight, increased sense of independence that may promote experimenting with dieting (food restriction, use of supplements and fad diets), and changes to daily schedule that affect eating and exercise habits ("pulling all-nighters" to study for exams or complete course projects).

Emergence of dieting among girls is most prevalent at 13 and 14 years of age and remains prevalent throughout adulthood [5]. Dieting has been reported among normal and underweight individuals, in addition to those who are overweight [6]. Despite the finding that dieting to control weight is often times ineffective; dieting remains popular among females [7]. When compared to college males, college females are more likely to actively diet, place high importance on appearance and the benefits of maintaining an ideal weight, and engage in unsafe dieting [8].

Given our culture, which suffers from a pathological emphasis on weight as a measure of a woman's worth, and given the continuing epidemic in our society of disordered eating, there are significant problems stemming from the idea of the "Freshman 15". The first problem is the assumption that excessive weight gain is inevitable during the college years. Second, for the many females already struggling with disordered eating, the construct of the "Freshman 15" becomes a terrifying probability that exacerbates an already difficult problem. For example, Patton [9] reported that girls with a history of severe dieting were 18 times more likely to develop eating disorders than girls with no history of dieting, and girls with a history of moderate dieting were five times more likely to develop eating disorders than girls who did not diet. Third, even for the college females who may not have significant issues with food or weight, the idea of the "Freshman 15" can play a significant role in solidifying the oppressive ideathat a female must be thin in order to qualify for success and happiness in our society. Such mentality can contribute significantly to the development of disordered eating throughout college.

At the core of what makes the "Freshman 15" concept so problematic is the reduction of female students to a single-dimension: body type. Given the myriad life issues that are relevant to women at the onset of the college years, to have a concept referring to the single superficial issue of body weight be so widespread and so influential is indicative of a larger cultural pathology. What is needed is a more aggressive message to the culture that contradicts this reductionistic message.

In this regard, there remain gaps in the research related to dieting among college females. Namely, do normal weight individuals diet differently from those who are overweight or obese, and are there dieting practices used by females that can be adapted to promote a healthy body weight? Because it is well recognized that females diet, this study seeks to determine the dieting practices used among normal, overweight, and obese college females (do they diet differently) and identify dieting practices that could be pursued to help these females more appropriately achieve and maintain a healthy body weight.

Of concern to health educators is the suggestion that college females practice diet and health behaviors that contradict the 2005 dietary guidelines for Americans [10]. For example, physical exercise plays a role in weight management [10,11]. In an executive summary regarding treatment of overweight and obesity in adults, the National Heart, Lung, and Blood Institute in cooperation with the National Institutes of Diabetes and Digestive and Kidney Diseases report that an increase in physical activity is an important component of weight loss therapy and that sustained physical activity is helpful in prevention of weight regain [11]. Importantly, the Dietary Guidelines recommend adults engage in 60 to 90 minutes of daily moderate- to vigorous-intensity physical activity [10]. It is questionable if college females adhere to the public health message of higher intensity and longer duration physical activity to promote a healthy body weight [10,11]. The relationship between smoking and weight concerns has been explored. Potter et al [12] reported in a comprehensive review of smoking among adolescents that dieting behaviors, disordered eating symptoms, and general weight concerns were related to smoking among females.

The purpose of this study was to determine differences in dieting practices, weight perceptions, and body composition of normal weight, overweight, and obese female college students. The research questions under investigation seek to first, determine body composition, weight perceptions, and physical activity differences of normal weight, overweight, and obese college females. Second, identify perceived sources of pressure to be a certain weight among these groups. Third, identify weight loss behaviors ever used to consciously lose or control weight among groups. Fourth, identify the most prevalent weight control products and commercial diets used by female college students.

## Methods

### Design and sample

The study is a quasi-experimental design. The sample of convenience included students from five introductory nutrition courses taught by two instructors and invited to participate during the spring semester of 2005. The total sample recruited included 230 females, which represented 51% of all females who were enrolled in introductory nutrition courses spring 2005. Overall, 208 female participants completed the study, which was a 90% participation rate for females in the nutrition courses surveyed. The final sample size was 185, as 23 were excluded for the following reasons: age greater than 24 years (n = 14), BMI less than 18.5 kg/m^2 ^(n = 7), and reported pregnancy (n = 2).

Course enrollment ranged from 49 to 100 students. A scripted description of the study (read by the instructor at the beginning of a class period which highlighted the study protocol) and an electronic course announcement posted on Blackboard were the means used to inform students about the study. For the purpose of this study Blackboard is defined as a web-based education platform that aids course delivery; students are accustomed to checking Blackboard frequently for course information. Extra credit was available to students who completed the study. Males, females greater than 24 years of age, and females reporting current pregnancy were excluded from analysis.

### Data collection and research instruments

Data collection occurred on two occasions. During the first meeting, students completed a survey regarding dieting practices, weight perceptions, and level of physical activity participation. During the second meeting, body composition assessments were completed. The university-affiliated institutional review board approved the project (University and Medical Center Institutional Review Board number 04–0487), and the project was carried out in compliance with the Helsinki Declaration. Signed informed consent was obtained from all participants.

Prior to survey administration, one of two research assistants met with students during a class period, using a script to describe the study and provide instruction for completing the survey. The key points of the script were to provide uniform instruction for completing the study survey, encourage participants to answer all questions completely and truthfully, and to remind participants of the body composition assessment schedule. The survey took approximately 15 minutes to complete.

The survey included a dieting practices questionnaire that assessed demographic, dieting practices, weight perceptions, and perceived sources of pressure to be a certain weight, and a 30-day physical activity recall. The dieting practices questionnaire was developed by a Registered Dietitian and reviewed for content validity by two Registered Dietitians who practice in eating disorder treatment and nutrition counseling of college students. The questionnaire was adapted from one developed by Calderon et al [13] that investigated dieting practices among high school students. As a measure of physical activity, a 30 day self-report measure developed by Jackson et al [14] was used. To complete this measure, participants rate how active they have been over the past 30 days on a scale ranging from zero to seven. In general, scores of zero and one indicate that the person does not participate in regular activity. Participants who score two or three participate in some moderate activity whereas those who score four or above participate in some vigorous exercise. Baumgartner et al [15] reported that values four or above generally indicate that a person participates in physical activity on a regular basis. Regular activity participation, particularly vigorous exercise, often results in increased aerobic capacity. In support of the scale's validity, researchers have found that self-reported physical activity was a significant predictor of VO_2 _max [15].

To pilot test the survey, a small sample of 12 undergraduate female students completed the questionnaires. Minor syntax and formatting modifications were made to the dieting practices questionnaire based on their responses.

On the second occasion, participants reported to the lab for anthropometric measurements. Females with the first letter of their last name A to L reported after completing the second exam in their respective nutrition course, females with last name M to Z after the third. The lab was organized into two measurement stations that were separated by privacy screens. Weight and height were measured at the first station, circumference (waist and hip) and skinfold thickness (triceps, biceps, and thigh) measurements were obtained at the second station. Four research assistants (two for each station) were trained by a single anthropometrist. The same research assistant took all measurements for that "station" on each participant. Weight was measured to the nearest 0.1 kg (Tanita body composition analyzer, Arlington, IL), height (Seca portable height stadiometer, Leicester, England) and circumferences (non-flexible tape measure) to 0.1 cm, and skinfolds to 0.1 mm (Harpenden skinfold caliper, Vital Signs model 68875, Country Technology, Inc., Gays Mills, WI) using American College of Sports Medicine [16] procedures. Shoes, and as much clothing as was socially acceptable to the participant, were removed preceding body composition measurements. Participants were classified according to body mass index (BMI); BMI 18.5 to 24.9 kg/m^2 ^were classified as normal weight, 25 to 29.9 kg/m^2 ^as overweight, and 30 kg/m^2 ^or greater as obese [17].

### Data analysis

Analyses were performed using JMP IN® software [18]. Descriptive statistics included means, standard deviations, and frequency. Subsequent data analysis involved Pearson *X*^2 ^and one-way analysis of variance with comparison for all pairs that were significantly different using Tukey-Kramer honestly significant difference test. A significant level of .05 was used for statistical analysis.

## Results

Overall, 208 female participants completed the study, which was a 90% participation rate for females in the nutrition courses surveyed. However, 23 were excluded for the following reasons: age greater than 24 years (n = 14), BMI less than 18.5 kg/m^2 ^(n = 7), and reported pregnancy (n = 2). BMI less than 18.5 kg/m^2 ^is classified by the Center for Disease Control and Prevention [17] as an underweight condition. The final sample size was 185. Mean age of participants was 19.7 years (SD = 1.4). A majority (78%) of participants were White, 18% were Black, and 4% were American Indian, Asian, or Hispanic. Participants were predominantly freshmen and sophomores (70%), as expected because they were recruited from introductory nutrition courses.

Regarding question one, body composition, weight perceptions, and physical activity of normal weight, overweight, and obese groups are reported in Table 1. Despite significant differences among groups for weight, F(2, 166) = 171, p < .01, BMI, F(2, 166) = 272, p < .01, body fat, F(2, 166) = 96, p < .01, and waist-to-hip ratio, F(2, 166) = 53, p < .01, mean physical activity was similar among groups, F(2, 165) = 0.5, p = .61. Significant differences were found among groups for perceived healthy weight, F(2, 163) = 76, p < .01, and attractive weight, F(2, 160) = 74, p < .01, compared to current weight. Mean perceived healthy weight was 5% (normal weight), 13% (overweight), and 23% (obese) lower than current weight. Mean perceived attractive weight followed the same trend, and was 6%, 15%, and 26% lower than current weight for normal weight, overweight, and obese groups, respectively. All groups perceived natural weight to be greater than current weight, meaning if no attempt was made to control weight then weight would be 2% to 6% greater than current weight. The majority (80%) of participants reported using physical activity to control their weight. However, 32% did not participate in regularly programmed recreation, sport, or heavy physical activity, and only 19% of participants (21% normal weight, 21% overweight, and 5% obese groups) reported spending over 3 hours per week in vigorous aerobic activity.

**Table 1:** Mean body composition, weight perceptions, and physical activity among normal weight, overweight, and obese female college students.

	Weight classification		
	
	Normal weight	Overweight	Obese
	n = 113	n = 35	n = 21
Variable	M ± SD	M ± SD	M ± SD
Body composition			
Weight (pounds)	130^a ^± 13	160^b ^± 18	213^c ^± 39
BMI (kg/m^2^)	21.9^a ^± 1.8	27.3^b ^± 1.3	35.3^c ^± 5.7
Body fat (%)	28^a ^± 4	33^b ^± 4	38^c ^± 3
Waist-to-hip ratio	0.72^a ^± 0.01	0.76^b ^± 0.05	0.83^c ^± 0.07
Weight perceptions			
Perceived healthy weight (pounds)	123^a ^± 10	138^b ^± 17	164^c ^± 26
Perceived attractive weight (pounds)	122^a ^± 11	136^b ^± 17	158^c ^± 29
Perceived natural weight (pounds)	138^a ^± 18	169^b ^± 25	216^c ^± 42
Perceived healthy weight (% of current)	95^a ^± 7	87^b ^± 6	77^c ^± 6
Perceived attractive weight (% of current)	94^a ^± 7	85^b ^± 6	74^c ^± 8
Perceived natural weight (% of current)	106 ± 9	106 ± 10	102 ± 2
Physical activity	4.1 ± 2.5	3.8 ± 2.5	3.5 ± 2.5

Note: Means in the same row having letters that do not share subscripts differ at *p *< .01 in the Tukey-Kramer honestly significant difference test. The higher the physical activity score, the greater intensity and amount of reported exercise over the past 30 days using a scoring system of 0 (do not participate regularly in planned exercise) to 7 (spend over 3 hours weekly in heavy exercise).

Concerning question two, 83% of participants reported ever consciously trying to lose or control their weight, including 80% of normal weight, 91% of overweight, and 86% of obese participants. Mean age when dieting was initiated was 15.7 years (SD = 2.3), which was similar among groups, F(2, 137) = 1, p = .30. Fifty eight percent of participants reported pressure to be a certain weight; the primary sources were self (54%), media (37%), and friends (32%). Frequency distributions of "yes" response to these sources were similar among groups (see Table 2). Other sources of pressure included significant other (reported as a source of pressure by 13% of participants), colleagues at work (5%), other athletes (3%), coach (2%), and family members (2%).

**Table 2:** Perceived sources of pressure to be a certain weight among normal weight, overweight, and obese female college students.

Variable	Frequency "yes" response (%)	*X*^2^	p (weight status)
Self		2.5	.29
Normal weight^a^	50		
Overweight^b^	62		
Obese^c^	43		
Media		0.9	.63
Normal weight^a^	39		
Overweight^b^	34		
Obese^c^	29		
Friends		3.4	.18
Normal weight^a^	31		
Overweight^b^	37		
Obese^c^	14		

Note: *X*^2 ^(2, *N *= 166).

^a^n = 113; ^b^n = 35; ^c^n = 21.

Referring to question three, 15 dieting behaviors were assessed to determine if use differed among normal weight, overweight, and obese participants. Nine of the 15 behaviors had large enough sample size of "yes" response to analyze frequency distribution differences among groups; these are reported in Table 3. The five most common behaviors used by all participants were exercising (80%), eating or drinking low fat or fat free versions of foods/drinks (59%), consciously eat less than you want (51%), eating or drinking sugar free versions of foods/drinks (43%), and count calories (40%). Eating less than you want and use of artificial sweeteners were the only behaviors for which frequency of use was significantly different among groups (see Table 3). The other behaviors assessed, which had low frequency of use reported, included not eating foods with high glycemic index (used by 4% normal weight, 6% overweight, and 0% obese), smoke cigarettes (8%, 14%, and 5%), use laxatives after you eat (2%, 6%, and 5%), vomit after you eat (4%, 6%, and 5%), skip lunch (10%, 9%, 10%), and skip dinner (4%, 9%, 0%). Explicit maladaptive weight loss practices included smoking cigarettes (used by 9% of all participants), vomiting (5%) and use of laxatives (3%) after eating.

**Table 3:** Weight loss behaviors ever used to consciously lose or control weight of normal weight, overweight, and obese female college students.

Variable	Frequency "yes" response (%)	*X*^2^	p (weight status)
Consciously eat less than you want		10.1	< .01
Normal weight^a^	44		
Overweight^b^	57		
Obese^c^	81		
Count grams of fat		1.0	.60
Normal weight^a^	23		
Overweight^b^	31		
Obese^c^	24		
Count net carbs		2.3	.32
Normal weight^a^	18		
Overweight^b^	17		
Obese^c^	5		
Count calories		0.2	.89
Normal weight^a^	39		
Overweight^b^	43		
Obese^c^	43		
Eat or drink low fat or fat free versions of foods/drinks		2.9	.24
Normal weight^a^	57		
Overweight^b^	71		
Obese^c^	52		
Eat or drink sugar free versions of foods/drinks		2.2	.33
Normal weight^a^	43		
Overweight^b^	49		
Obese^c^	29		
Exercise		2.2	.33
Normal weight^a^	77		
Overweight^b^	89		
Obese^c^	81		
Use artificial sweeteners			
Normal weight^a^	31	6.3	.04
Overweight^b^	31		
Obese^c^	5		
Skip breakfast			
Normal weight^a^	27	4.9	.09
Overweight^b^	40		
Obese^c^	48		

Note: *X*^2 ^(2, N = 166).

^a^n = 113; ^b^n = 35; ^c^n = 21.

In terms of question four, over-the-counter meal replacement drinks were used by 35% of participants, supplements (Hydroxycut, Stacker II, Xenadrine, CortiSlim, Trim Spa, Hot Rox) by 26%, meal replacement bars by 18%, dieter's tea, green tea, and green tea pills by 11%, and chromium picolinate by 3%. Physician prescribed weight loss pills were used by 3% of participants. In examining commercial diets, the most popular ones were Atkins or South Beach (20%) and Weight Watchers® (11%). Subway, Sugar Busters, and The Zone diet combined had been used by 7% of participants.

## Discussion

The purpose of this study was to investigate dieting practices, weight perceptions, and body composition among normal weight, overweight, and obese college females. Findings from this study support the general belief that dieting by college females is a common weight management strategy, irrespective of weight status. Abraham and O'Dea [19] reported that females as young as 12 years of age had tried to lose weight, including 44% who dieted using food restriction and 78% who exercised to lose weight. Findings from the present study indicate that females continue to diet through college. Calderon et al [13] reported a greater prevalence of dieting among overweight (67% prevalence) versus at risk for overweight (25%) high school females, which is in contrast to the findings from the present study. We found 83% of college females reporting ever consciously trying to lose or control their weight, with similar prevalence between normal weight, overweight, and obese participants. The discrepancy between findings could be due to how dieting is defined by researchers and interpreted by study participants. Dieting strategies have become so "main stream" in our society, that one may not be aware, unless explicitly stated, behaviours that are being used to consciously lose or control ones' weight. For example, someone may have been drinking diet sodas to control/lose their weight for so many years that they do not recognize this behaviour as a weight loss strategy unless it is pointed out to them. We classified dieting as any method of food restriction, use of supplements, or behaviours used to control one's weight. Although our questionnaire was adapted from the survey of Calderon and colleagues [13], we expanded upon behaviours and methods marketed for weight control that were popular at the time our study was conducted, including counting net carbs, eating/drinking sugar free versions of foods/drinks, avoiding foods with high glycemic index, and use of artificial sweeteners. Furthermore, a limitation of the study is that being a cross-sectional study design, we do not know if there exists a causal relationship between dieting and weight control. Longitudinal studies would be the best way to measure if dieting frequency among females and methods of dieting choices change throughout the lifespan and if there exists a causal relationship between dieting and weight control.

We found females perceive healthy and attractive weights to be lower than current weight, and that media influence contributes pressure to be a certain weight. Klesges et al [8] reported that college females are more likely than male counterparts to engage in healthy and unhealthy physical activity and food restriction behaviours and place higher importance on appearance benefits of maintaining an ideal body weight. Of great concern are the findings of Bacon et al [20] that indicated a significant negative correlation between bone mineral content and number of times on a weight loss diet among obese adult females. Additionally, Gingras et al [21] reported that adult female chronic dieters had low body satisfaction and suggested that it is important to address body image dissatisfaction with chronic dieting to improve health, regardless of body size of the dieter. Clearly, chronic dieting can have physical and emotional ramifications.

Most important, many of the weight loss practices reported to be used by participants of the current study, if used appropriately, could promote healthy weight loss and be used long term to maintain a healthy weight. For example, the majority (80%) of participants reported using physical activity to control their weight. However, 32% did not participate in regularly programmed recreation, sport, or heavy physical activity, which is similar to the Behavioral Risk Factor Surveillance system finding that 15% to 34% of adults reported no leisure-time physical activity [22]. Holcomb et al [23] reported that body mass index, body fat, and waist-to-hip ratio were significantly lower as physical activity increases among premenopausal females. The researchers suggested that ability to increase daily physical activity to minimize fat accumulation could be a strong incentive to become more physically active. Strategies to improve nutrient intake and physical activity to promote health and weight control among females are warranted.

The current study makes an important contribution to the literature by narrowing the gap in research related to dieting among females. Namely, do normal weight individuals diet differently from those who are overweight or obese, and are there dieting practices used by females that can be adapted to promote a healthy body weight? Outcomes of interest to the health educator include: First, dieting practices are prevalent among female college students, regardless of body weight; Second, there were a number of dieting practices used that could promote effective weight loss and healthy weight maintenance if employed appropriately; Third, although many dieters stated they used physical activity for weight loss, the majority who used this method were not meeting physical activity goals for weight loss; and Fourth, the most prevalent explicit maladaptive weight loss behavior identified was smoking cigarettes and the most unhealthy dieting practice was skipping breakfast.

## Conclusion

These findings suggest that health educators should promote education and intervention strategies for females that encourage appropriate weight control practices and dispel unhealthy and ineffective weight loss myths (using laxatives and skipping breakfast are effective weight control methods). Implications from this study are numerous. Female college students, regardless of weight status, would benefit from open discussions with health educators to identify healthy and unhealthy dieting practices they use. Throughout these discussions, females could identify healthy dieting practices that could be expanded upon to promote a healthy weight status and recognize the health consequences associated with using unhealthy dieting practices. Because this study involved only female college students at a state university the implications discussed here are subject to replication of the results. Beyond replication with other participants, researchers can consider how to effectively target specific dieting practices among females to promote healthy eating and exercise to promote and maintain a healthy weight. Future research should identify which dieting practices dieters report most content with and believe are most successful to promote short- and long-term weight management.

## Competing interests

The author(s) declare that they have no competing interests.

## Authors' contributions

BMM conceived of the study, participated in its design, performed the statistical analysis, and drafted the manuscript. TAR participated in the design of the study and drafted the manuscript. VGA drafted the manuscript. JLS and MBD participated in coordination and data collection and helped to draft the manuscript. All authors read and approved the final manuscript.
